# Family planning services financing in the West African Economic and Monetary Union countries: Trend analysis from 2013 to 2021

**DOI:** 10.4102/jphia.v17i1.1690

**Published:** 2026-07-14

**Authors:** Danielle Yugbaré Belemsaga, Simon T. Nassa, Yann Tapsoba, Kadari Cissé, Désiré L. Dahourou, Henri G. Ouedraogo, Seni Kouanda

**Affiliations:** 1 Department of Biomedical and Public Health, Institute for Research in Health Sciences (IRSS), National Center for Scientific and Technological Research (CNRST), Ouagadougou, Burkina Faso; 2 Ministry of Health, Ouagadougou, Burkina Faso; 3 Independent Consultant, Ouagadougou, Burkina Faso

**Keywords:** health expenditure, family planning, GHED, West African Economic and Monetary Union (WAEMU) countries, financing, trend analysis

## Abstract

**Background:**

Access to high-quality family planning (FP) services is crucial for addressing unintended pregnancies and maternal mortality. There is uncertainty about health expenditures, especially FP services, by financing sources.

**Aim:**

The study aims to conduct key analyses on health and reproductive health expenditures in the West African Economic and Monetary Union (WAEMU).

**Setting:**

This analysis was performed across the eight WAEMU countries (Benin, Burkina Faso, Côte d’Ivoire, Guinea-Bissau, Mali, Niger, Senegal and Togo) from 2013 to 2021.

**Methods:**

We conducted a secondary data analysis using the Global Health Expenditures Database. The variables were the current health expenditures, reproductive health expenditures, and FP expenditures by funding source (National General Government, External Funding Sources, and National Private Sector). We performed a trend and funding-source analysis in purchasing power parity in United States dollars.

**Results:**

Current expenditures allocated to health, reproductive, and FP services increased in recent years, growing by 4.6%, 5.9%, and 2.7% per year, respectively, in the region. The evidence is strong that countries have commitments to improving health and strengthening access to reproductive health services, especially modern contraceptives. However, FP funding is still dependent on external resources. In most countries, including Benin, Burkina Faso, Guinea- Bissau, Mali, and Senegal, more than half of FP expenditures are funded by external resources, whereas Côte d’Ivoire and Niger rely heavily on government resources. In Togo, the main FP funding sources are the domestic private resources.

**Conclusion:**

The significant dependence of Public Finance management systems on external funding, with the government’s low contribution, may undermine the sustainability of FP programmes in these countries.

**Contribution:**

The evidence suggests increasing domestic resource mobilisation for FP through integrated co-financing mechanisms and advocacy for FP reprioritisation within governments’ health budgets, and ensuring efficient and equitable allocation of existing resources through resource reprogramming based on the country background, national priorities, and high-impact interventions.

## Introduction

Access to high-quality family planning (FP) services is fundamental to improving reproductive health and general well-being, particularly in developing countries.^1,2^ These services not only reduce the risks of unwanted pregnancies and maternal complications, but also empower women by giving them greater control over their health and family life.^3^ Since the West African Economic and Monetary Union (WAEMU) countries, including Benin, Burkina Faso, Côte d’Ivoire, Guinea-Bissau, Mali, Niger, Senegal, and Togo,^4^ are facing several health challenges coupled with a shortfall of resources, access to planning family plays a critical role in reducing maternal and infant mortality, as well as in strengthening the socio-economic development. The WAEMU countries have an integrated macroeconomic and financial framework that promotes a harmonised management of public resources.^4^ This economic integration makes funding more predictable, a key factor in the success of FP programmes. The common framework with macroeconomic convergence criteria strengthens countries’ capacity to mobilise and allocate financial resources efficiently, including for public health programmes such as FP. The alignment of fiscal and budgetary policies at the WAEMU level facilitates the implementation of regional programmes and access to joint or external funding. West African Economic and Monetary Union budget priorities include sectors such as health, which can help to ensure regular and adequate funding for FP. Solidarity mechanisms within WAEMU enable member countries to benefit from support funds or share best practices, especially in public finance management.^4,5^ This solidarity is crucial in strengthening national FP systems, especially in countries with limited domestic resources.^6^

Nearly half of all pregnancies in low-income countries are unwanted.^1,3^ Gahungu identified an association between unmet needs for FP, health system, and socio-demographic determinants in Sub-Saharan Africa.^7^ The gaps between meeting contraceptive needs and funding are persistent in several regions and countries, including the WAEMU.^8^ There is very little scientific evidence on health spending in these countries, especially the spending on FP services in the WAEMU countries. Understanding the financial dynamics behind FP services is essential for informing policies aimed at strengthening coverage of quality FP services.

National Health Accounts (NHA) are essential for understanding and optimising financial flows within countries’ healthcare systems.^9^ They are used to evaluate the health financing system and to identify the most efficient interventions to improve the health of the population. Their use helps to optimise the allocation and transparent management of resources.^10^

To strengthen the sustainability and effectiveness of FP services, this paper aimed to analyse spending allocated to FP services, with a focus on WAEMU countries, from 2013 to 2021. It consists of a cross-country analysis of the trends and the structure of health spending allocated to FP services.

## Research methods and design

### Study setting

We conducted our analysis in WAEMU countries (Benin, Burkina Faso, Côte d’Ivoire, Guinea-Bissau, Mali, Niger, Senegal, and Togo) from 2013 to 2021.

### Study design

We conducted a secondary data analysis from the World Health Organization (WHO) Global Health Expenditure Database, completed with the NHA data (Production Tool Studies).

### Conceptual framework

**FIGURE 1:**
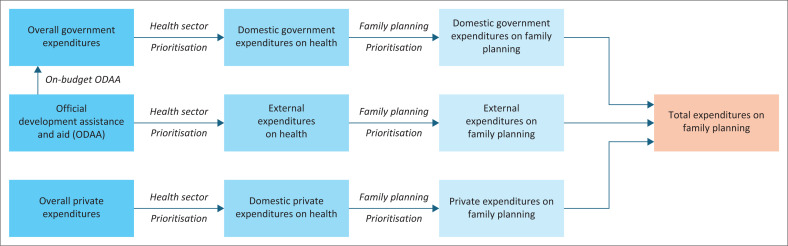
Connecting family planning financing to health sector expenditure.

Family planning expenditures are a component of reproductive health expenditures and depend on three financing sources: own government resources, domestic private-sector resources, and external funding (Figure 1).

Domestic government expenditures on FP are driven by: (1) overall government expenditures (including on-budget official development assistance and aid (ODAA); (2) the health sector prioritisation level in overall government expenditures – proxied through domestic government expenditures on health as a share of overall government expenditures; and (3) the level of FP prioritisation in domestic government health expenditures – proxied through domestic government expenditures on FP as a share of domestic government expenditures on health.

Domestic private expenditures on FP are driven by: (1) the envelope level of overall private expenditures; (2) the health sector prioritisation level in overall private expenditures – proxied through domestic private expenditures on health as a share of overall private expenditures; and (3) the level of FP prioritisation in domestic private health expenditures – proxied through domestic private expenditures on FP as a share of domestic private expenditures on health.

The external expenditures on FP are driven by (1) the ODAA, (2) the health sector prioritisation level in ODAA – proxied through external expenditures on health as a share of ODAA – and (3) the level of FP prioritisation in external health expenditures – proxied through external expenditures on FP as a share of external expenditures on health.

### Description of data source

The GHED is a database that compiles internationally validated information, enabling reliable comparisons of health expenditures across countries according to the international methodology of the 2011 System of Health Accounts (SHA).^11^ The SHA data, produced by countries, are from primary and secondary sources.^12^ The health accounts measure health expenditures and resource flows by sources, functions of care, providers, factors of production, and both current and capital spending in health.^9,12^ The methodology adopted by the WHO aims to comply with international standards for the production of indicators, to ensure rigorous comparability between countries.^9^

We extracted several health expenditure indicators from the GHED and NHA, focusing on all WAEMU countries (Benin, Burkina Faso, Côte d’Ivoire, Guinea-Bissau, Mali, Niger, Senegal and Togo) over 2013-2021.

Country data on FP expenditures have been available in different time dimensions. Data for Burkina Faso, Niger, and Togo are available from 2013 to 2021, while data for Côte d’Ivoire and Senegal are available from 2013 to 2020. Mali data are available from 2013 to 2020, but there are no data on domestic government expenditures on FP from 2017 to 2020. Guinea–Bissau data are available from 2018 to 2021, but there are no data on domestic private spending in the FP, while Benin has data from 2013 to 2018.

### Study variables

The extracted study variables include NHA indicators such as current health expenditure (CHE), reproductive health expenditure, and FP expenditure by funding source (National General Government, External Funding Sources, and National Private Sector), Appendix 1 (key definitions), and Appendix 2 (method of measurement).

All health, reproductive health, and FP spending indicators focused on the current purpose. They were expressed in purchasing power parity (PPP) terms in United States dollars (USD, $) to account for country-level disparities in purchasing power and to ensure consistent cross-country benchmarking, without adjustment for inflation. However, since all the countries considered belong to the same economic and financial area, WAEMU has relatively uniform inflation.

### Data analysis

We used the health expenditure data by country and year without any changes. In the descriptive section of our analysis, we presented the trend in sexual and reproductive health expenditures, the proportion of current health expenditures (CHEs), and FP service expenditures, by WAEMU countries, by year. We finally describe the temporal trend in FP service expenditures by source of funding, by WAEMU country, and by year.

### Ethical considerations

This article followed all ethical standards for research without direct contact with human or animal subjects.

## Results

### Current health expenditure in purchasing power parity United States dollar by West African Economic and Monetary Union countries and by year from 2013 to 2021

**FIGURE 2:**
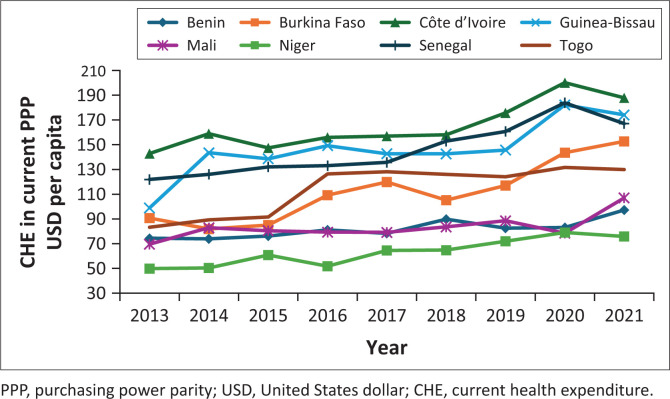
Current health expenditure in purchasing power parity United States dollars by West African Economic and Monetary Union countries by year from 2013 to 2021.

In WAEMU countries, CHEs significantly increased from 2013 to 2021. The regional average expenditures increased by 4.6% per year, changing from $91.40 PPP USD per capita in 2013 to $136.50 PPP USD per capita in 2021. This growth reflects increasing efforts by governments and donors to improve health status in the region. Most countries have gradually increased their spending (Figure 2).

### Reproductive health expenditures in million purchasing power parity United States dollar and as a share of current health expenditures across West African Economic and Monetary Union countries over 2013–2021

Reproductive health expenditures significantly increased from 2013 to 2021 (Table 1). The regional average expenditure on reproductive health changed from $9.3 million PPP USD in 2013 to $15.5m PPP USD in 2021, representing an average annual increase of 5.9% and reflecting a growing commitment to improving the reproductive health of people in the region. A granular analysis highlights significant variations across countries. In 2013, Niger recorded the highest reproductive health expenditures, estimated at $11m PPP USD, representing 21.7% of CHEs. However, these amounts have fluctuated, stabilising at around $8m PPP USD in 2021, representing 11.1% of CHEs.

**TABLE 1:** Reproductive health expenditures in million purchasing power parity United States dollar and as a share of current health expenditures across West African Economic and Monetary Union countries over 2013–2021.

Countries	Year																	
	2013		2014		2015		2016		2017		2018		2019		2020		2021	
	Amount ($)	%	Amount ($)	%	Amount ($)	%	Amount ($)	%	Amount ($)	%	Amount ($)	%	Amount ($)	%	Amount ($)	%	Amount ($)	%
Benin	10.0	13.1	9.0	11.7	9.0	11.7	9.0	11.3	9.0	11.4	10.0	11.2	-	-	-	-	-	-
Burkina Faso	8.0	8.6	8.0	9.7	9.0	10.2	13.0	12.0	19.0	16.1	16.0	15.4	17.0	14.5	18.0	12.3	24.0	15.9
Côte d’Ivoire	10.9	7.6	7.6	4.8	17.4	11.8	19.2	12.3	17.1	10.9	16.4	10.4	10.9	6.2	12.3	6.1	-	-
Guinea-Bissau	-	-	-	-	-	-	-	-	-	-	15.0	10.4	9.0	6.3	5.0	2.6	7.0	3.8
Mali	5.0	7.9	9.0	11.4	9.0	11.3	8.0	10.2	2.0	1.9	10.0	12.4	14.0	15.7	1.0	1.3	-	-
Niger	11.0	21.7	8.0	16.1	8.0	13.9	8.0	15.2	6.0	9.4	8.0	12.0	5.0	7.1	5.0	6.0	8.0	11.1
Senegal	11.0	9.1	22.0	17.1	18.0	13.6	18.0	13.7	26.0	19.3	25.0	16.1	30.0	18.8	21.0	11.6	-	-
Togo	9.0	11.3	10.0	11.3	11.0	11.8	14.0	10.9	12.0	9.5	13.0	10.0	10.0	8.4	30.0	23.1	23.0	17.9

Burkina Faso has shown strong growth in reproductive health expenditures, rising from $8m PPP USD in 2013 to $24m PPP USD in 2021, representing 8.8% and 15.9% of CHEs, respectively. Regarding Côte d’Ivoire, the country posted reproductive health expenditures of $10.9m PPP USD in 2013, followed by a slight fall to $7.6m PPP USD in 2014, before peaking at $19.2m PPP USD in 2016, then dropping to $12.3m PPP USD in 2020. Senegal has also experienced variation, with reproductive health expenditures rising from $11m PPP USD in 2013 to a peak of $26m PPP USD in 2017, before falling back to $21m PPP USD in 2020. By contrast, Mali, Togo, and Guinea–Bissau have more modest spending levels, with Togo peaking at $30m PPP USD in 2020 before falling to $23 PPP USD in 2021. In Mali, reproductive health expenditures stagnated at around $8m to $10m PPP USD over 2014–2018, peaking at $14m PPP USD in 2019. In Guinea–Bissau, spending only started in 2018 at $15m PPP USD, representing 10.4% of its budget, before dropping to $5m PPP USD in 2020.

### Contraceptive management (family planning) expenditures in million purchasing power parity United States dollar and as a share of reproductive health expenditures across West African Economic and Monetary Union countries over 2013–2021

Contraceptive expenditures in WAEMU countries show a general upward trend from 2013 to 2021 (Table 2). The average expenditure on contraceptive care in the region increased from $1.4m PPP USD in 2013 to $1.7m PPP USD, representing an average annual increase of 2.7% and illustrating a growing commitment to FP in WAEMU countries. Additional granular analysis highlights some disparities across countries. Côte d’Ivoire stands out with spectacular jumps in 2017 and 2020 ($7.6m PPP USD), higher than in other years. Burkina Faso shows an increasing trend, rising from $1.2m PPP USD in 2013 to $3.3m PPP USD in 2021. Guinea–Bissau presents modest figures from 2018, peaking at $1m PPP USD in 2021. Togo has seen wide variations, peaking at $3.5m PPP USD in 2016 before dropping to $0.4m PPP USD in 2021. Niger and Senegal show a steady increase in expenditure, with a peak in 2017 for Niger ($3.7m PPP USD) and sustained levels for Senegal (peaking at USD $3.4 PPP in 2019).

**TABLE 2:** Contraceptive management (family planning) expenditures in million purchasing power parity United States dollar and as a share of reproductive health expenditures across West African Economic and Monetary Union countries over 2013–2021.

Countries	Year																	
	2013		2014		2015		2016		2017		2018		2019		2020		2021	
	Amount ($)	%	Amount ($)	%	Amount ($)	%	Amount ($)	%	Amount ($)	%	Amount ($)	%	Amount ($)	%	Amount ($)	%	Amount ($)	%
Benin	0.6	5.7	1.4	16.4	2.4	26.6	2.9	31.1	2.7	30.2	3.4	33.4	-	-	-	-	-	-
Burkina Faso	1.2	15.5	1.7	20.8	2.1	24.5	2.7	20.3	1.9	9.9	2.2	13.5	1.5	8.8	3.1	17.5	3.3	13.7
Côte d’Ivoire	0.6	5.2	1.6	20.9	2.9	16.4	3.5	18.1	7.6	44.2	4.1	25	4.6	42.7	7.6	62.0	-	-
Guinea-Bissau	-	-		-	-	-	-	-	-	-	0.8	5.7	0.8	8.2	0.9	18.7	1.0	15.8
Mali	0.6	11.5	2.6	27.1	2.0	22.1	1.6	20.1	0.3	22.1	2.3	22.1	1.2	8.3	0.4	36.1	-	-
Niger	3.4	31.7	1.8	22.4	1.6	19.2	1.4	18.2	3.7	60.9	1.6	21.1	1.8	35.0	2.0	43.0	2.2	26.3
Senegal	1.0	4.8	3.0	13.1	2.0	9.0	2.0	11.0	2.0	8.2	2.0	7.7	3.4	11.1	2.0	11.3	-	-
Togo	2.1	22.0	3.0	29.6	2.7	24.9	3.5	25.7	2.8	23.5	3.4	27.3	2.5	24.3	1.3	4.3	0.4	1.8

### Contraceptive management (family planning) expenditures by funding sources across West African Economic and Monetary Union countries, from 2013 to 2021

Contraceptive expenditures in WAEMU countries show disparities across funding sources (domestic government, external sources, and domestic private) between 2013 and 2021 (Table 3). The general trend shows that external funding plays a major role, but its importance varies across countries. Côte d’Ivoire has the lowest reliance on external financing, at only 12.83% in 2020, compared with countries such as Guinea–Bissau, Burkina Faso, and Mali, where external financing is dominant. Côte d’Ivoire and Niger managed to increase government contribution to FP expenditures. Burkina Faso has better mobilisation of domestic funds than Benin, but Niger shows impressive domestic fund mobilisation. Contribution from private domestic sources also varies, with substantial contributions in countries such as Togo, Niger, and Benin. However, private contributions remain low in some countries, such as Mali. In Senegal, FP expenditures have been primarily funded by external donors and the private sector. On average, external resources were the predominant funding source of contraceptives in more than half of the countries, including Benin (representing 54.8% of FP expenditures), Burkina Faso 65.6% of FP expenditures), Guinea–Bissau (representing 69.4% of FP expenditures), Mali (85% of FP expenditures), and Senegal (representing 56% of FP expenditures). In Côte d’Ivoire and Niger, the largest source of funding was external donors, who contributed 75.1% and 53.7% of FP expenditures, respectively, while FP services in Togo were essentially funded through domestic private resources, accounting for 58.5%.

**TABLE 3:** Contraceptive management (family planning) expenditures by funding sources across West African Economic and Monetary Union countries, over 2013–2021.

Countries	Indicators (%)	Year								
		2013	2014	2015	2016	2017	2018	2019	2020	2021
Benin	Domestic general government	13.17	3.76	2.09	1.43	1.49	1.24	-	-	-
	External sources of funding	50.87	78.13	42.35	51.76	48.45	57.38	-	-	-
	Domestic private	35.95	18.11	55.56	46.81	50.06	41.37	-	-	-
Burkina Faso	Domestic general government	11.02	14.61	4.28	0.00	6.31	23.69	2.05	14.79	16.78
	External sources of funding	83.11	71.79	47.74	87.03	49.24	29.73	73.49	72.80	75.75
	Domestic private	5.86	13.60	47.99	12.97	44.46	46.58	24.46	12.41	7.48
Côte d’Ivoire	Domestic general government	87.95	31.35	75.67	59.36	92.76	84.43	83.09	86.02	-
	External sources of funding	12.05	61.50	20.38	34.35	5.76	13.07	14.70	12.83	-
	Domestic private	0.00	7.15	3.95	6.29	1.48	2.50	2.21	1.15	-
Guinea-Bissau	Domestic general Government	-	-	-	-	-	23.30	24.53	42.90	31.83
	External sources of funding	-	-	-	-	-	76.70	75.47	57.10	68.17
Mali	Domestic general government	2.31	0.16	1.10	0.16	0.00	0.00	0.00	0.00	-
	External sources of funding	80.89	99.4	98.79	99.84	95.93	89.11	97.59	18.27	-
	Domestic private	16.80	0.45	0.11	0.00	4.07	10.89	2.41	81.73	-
Niger	Domestic general government	31.05	59.53	54.82	61.26	27.35	47.10	68.52	70.35	63.65
	External sources of funding	5.42	33.34	43.98	24.43	15.98	31.48	23.90	20.25	17.79
	Domestic private	63.53	7.13	1.20	14.31	56.67	21.42	7.58	9.40	18.56
Senegal	Domestic general government	5.59	3.51	7.58	11.81	11.99	14.45	7.80	13.16	-
	External sources of funding	24.11	80.42	63.11	62.00	64.72	59.29	71.46	22.61	-
	Domestic private	70.30	16.07	29.31	26.20	23.29	26.26	20.74	64.23	-
Togo	Domestic general government	21.98	15.27	22.41	8.59	20.53	17.08	14.65	5.77	12.70
	External sources of funding	7.69	30.94	3.82	7.51	5.23	18.31	2.66	88.38	70.03
	Domestic private	70.32	53.79	73.77	83.90	74.24	64.61	82.70	5.85	17.27

## Discussion

This article analysed trends in health and reproductive health expenditures across the eight WAEMU countries from 2013 to 2021, with a focus on FP.

The evidence indicates a general upward trend in health and reproductive health expenditures, highlighting strong country commitments to improving health status through resilient health systems capable of delivering high-quality reproductive health services. However, spending fluctuations varied across countries, reflecting disparities in countries’ economic and budget constraints for resource mobilisation, as well as in political priorities for addressing health needs and emergencies.

Since the FP2020 initiative, the results indicate efforts towards additional resource mobilisation for FP, as reflected in increased expenditures allocated to contraceptive management, except in Mali and Niger. Additionally, for most countries, FP funding has been depended on external resources, with low contributions from government resources.

These findings are aligned with the literature’s evidence, highlighting stagnated or even declining domestic health expenditures in low- and middle-income countries^10,13^ and quasi-absence of government contribution to health funding in the Democratic Republic of Congo (South Kivu).^14^

The country’s health financing system, dependent on external funding, sometimes fungible and volatile due to international geopolitics and the dynamics of external markets, may undermine the sustainability of health interventions^15,16,17,18^ and FP programmes, given the observed and expected shift in the allocation of external resources to the sector.

To strengthen access to FP services and commodities for reaching universal health coverage^19^ in the WAEMU region, the evidence from this study suggests domestic resource mobilisation. It is critical for countries to design co-financing models at the country ‘level’^20,21^ that need to be supported through technical support, political will, and financial commitments. For instance, the commitments of Nigerian States through a contributory financing model contributed to additional domestic resources for FP and reproductive health programmes^21^ and were sustained through subnational government ‘partnerships’.^22^

It is also important to implement advocacy actions for FP reprioritisation within the allocation of government ‘health budgets’^23^ with the support of working groups and networks engaged in the implementation of the FP 2020 initiative. Advocacy processes should also account for the Ouagadougou Partnership strategies, launched in 2011, to double the use of modern contraceptives by 2030, and support partners and civil society in the design, planning, implementation, monitoring, and dissemination of FP programmes.

Beyond resource mobilisation, it would also be essential to ensure efficient and equitable allocation of funding through FP resource reprogramming based on the national priorities, aligned with specific characteristics of each country^13^ and targeting the high-impact practices and interventions.^24^

### Limitations or constraints

Our study has some limitations. Because of missed data, (1) firstly, capital expenditures on health, reproductive health and FP have not been accounted for in the analysis, even though they are an important component of health expenditures; (2) secondly, data available from the GHED were only from year 2013 to 2021, so that there is no time series data for the last 10 years for the eight WAEMU countries, (3) The available FP data were not detailed.

## Conclusion

Our study assessed FP expenditures in WAEMU countries from 2013 to 2021 using GHED data. The results show that WAEMU countries should increase their domestic funding for FP services to reduce their dependence on external funding. They help to understand the dynamics of FP funding in WAEMU and improve the effectiveness of health policies in the region.

## Appendix 1

**TABLE 1-A1:** Key definitions.

Variables	Definition
Current expenditures on health, PPP USD	Current expenditures on health refer to government schemes and compulsory contributory health care financing schemes, voluntary health care payment schemes, household out-of-pocket payment, rest of the world financing schemes (non-resident) and unspecified financing schemes.
Reproductive health expenditures, PPP USD	Reproductive health expenditures refer to resources spent on maternal, perinatal, unspecified reproductive health conditions, and family planning.
Family planning expenditures, PPP USD	Family planning expenditures refer to resources spent on contraceptive management.
Domestic government expenditures on Family planning, PPP USD	Domestic government expenditures on family planning refer to the transfers from government domestic revenue (allocated to Family planning purposes) and social insurance contributions.
Domestic private expenditures on Family planning, PPP USD	Domestic private health expenditures refer to the private sector resources spent through compulsory and voluntary prepayment schemes, including other unspecified resources.
External expenditures on family planning, PPP USD	External expenditures on family planning refer to the transfers distributed by the government from foreign origin and direct foreign transfers (FS.7).

PPP, purchasing power parity; USD, United States dollar; FS, financial sources.

## Appendix 2

**TABLE 1-A2:** Method of measurement.

Indicator name	Method of measurement
Current Health Expenditure (CHE)	Government schemes and compulsory contributory healthcare financing schemes (HF.1) + Voluntary health care payment schemes (HF.2) + Household out-of-pocket payment (HF.3) + Rest of the world financing schemes (non-resident) (HF.4) + Unspecified financing schemes (n.e.c.) (HF.n.e.c)
Reproductive Health Expenditure (absolute and % of CHE)	Reproductive health expenditure and current health expenditure
Contraceptive expenditure (absolute and % of reproductive health)	Contraceptive expenditure and reproductive health Expenditure

HF, health care financing schemes (or health funding schemes)
